# A rare case of upper lip schwannoma: A case report with analysis of the histological, immunohistochemical and pathogenesis aspects

**DOI:** 10.1016/j.ijscr.2024.109445

**Published:** 2024-02-27

**Authors:** Rizqan Maulana, Muhammad Reza Pahlevi, Yosaphat Bayu Rosanto, Bramasto Purbo Sejati, Cahya Yustisia Hasan

**Affiliations:** a Oral and Maxillofacial Surgery Study Program, Faculty of Dentistry, Gadjah Mada University, Yogyakarta, Indonesia; b Department of Oral and Maxillofacial Surgery, Faculty of Dentistry, Gadjah Mada University, Yogyakarta, Indonesia

**Keywords:** Schwannoma, Upper lip, Tumor, Excision

## Abstract

**Introduction and importance:**

Schwannoma, a benign tumor originating from *Schwann* cells, is a rare case found intraorally. The tongue, palate and buccal mucosa are the most common sites of intraoral Schwannoma while it is very rarely found on the lips. Previous studies reported only twelve cases of Schwannoma on the upper lip. The etiology of Schwannoma is unknown, but in some literature, Schwannoma occurs due to a defect in the *NF2* gene. Management of Schwannoma is excision of the capsule. The prognosis is good, and the recurrency is low. This article reports a rare case of upper lip Schwannoma in adolescent and its management with its histological, immunohistochemical and pathogenesis aspects.

**Case presentation:**

A 16-years old female presented a painless, semi-solid, mobile lump on the upper lip measuring of approximately 1.5 × 3 cm that had similar color with the surrounding tissue. The lump appeared 7 years ago.

**Clinical discussion:**

Excision of the capsule and margins of the tumor. Histopathological examination showed a unique feature of Schwannoma, the *Verocay* bodies. Subsequent immunohistochemical examination of S100 protein showed a classic type of Schwannoma.

**Conclusion:**

Upper lip schwannoma is a very rare tumor, and this type of tumor cannot be distinguished from other benign soft tissue tumors based on clinical findings. Immunohistochemical results are in accordance with the Histopathological results for the final diagnosis of Schwannoma. Schwannoma can be used as a differential diagnosis in cases of lumps on the lips with sessile, similar color like surrounding tissue, painless, and movable features.

## Highlights

• Upper lip schwannoma in the adolescence
• A rare case of upper lip schwannoma
• Histology, immunochemistry and pathogenesis of upper lip schwannoma

## 1 Introduction

Schwannoma (Neurilemmoma) is a benign neurogenic tumor originating from *Schwann* cells with characteristics of slow growth, single, painless, and encapsulated [1,2]. *Schwann* cells are glial cells that surround the axons of neurons and form myelin sheaths, which play a role in the process of axon growth [2]. The case of Schwannoma was first reported by Verocay in 1910 and it was previously called a Neuronoma [3].

Schwannoma is a very rare tumor. Approximately 25–40 % of the Schwannomas occur in the head and neck, however, it is only 1 % in the intraoral area [4]. Intraoral Schwannoma is often found on the tongue, palate, and buccal mucosa, while lips and tongue base are the rare site of this lesion. The highest incidence occurs in the third and fourth decades of life [5,6].

Schwannoma on the lips was first discovered by Das Gupta in 1969 [7]. The management of Schwannoma is surgical excision up to the tumor capsule to avoid recurrence. Schwannoma has a good prognosis, with a low rate of recurrency if it is removed until the base of the tumor [8]. This article reports a rare case of upper lip Schwannoma in a 16-year-old female patient who underwent surgical excision with satisfactory results and no post-excision recurrence.

## 2 Case report

**Fig. 1:**
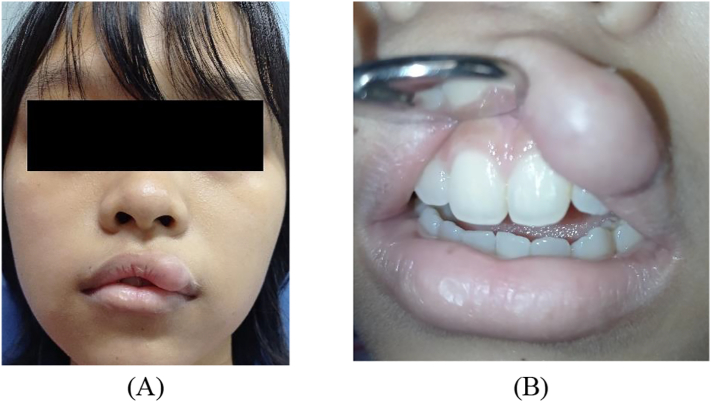
(A) Extraoral examination revealed swelling of the upper lip. (B) Upper lip had a sessile lump on the lip 1.5 × 3 cm, soft consistency, similar color with the surrounding tissue, painless, and movable.

This work has been reported in line with the SCARE criteria [9]. A 16-year-old female patient came to the Oral Surgery Clinic of Prof. Soedomo Dental Hospital, Universitas Gadjah Mada, Yogyakarta with a complaint of a lump on the left upper lip. The patient presented a good general condition and no disabilities. There was no history of systemic diseases, was not consuming routine medication, and no smoking habit. The lump appeared 7 years ago and grew slowly. Clinical examination revealed a sessile lump on the upper lip with a size of 1.5 × 3 cm, soft consistency, the color was similar to the surrounding tissue, painless, and movable (Fig. 1). The diascopy test was negative. The results of fine needle aspiration biopsy showed erythrocytes, lymphocytes, and macrophages. No signs of malignancy were found.

**Fig. 2:**
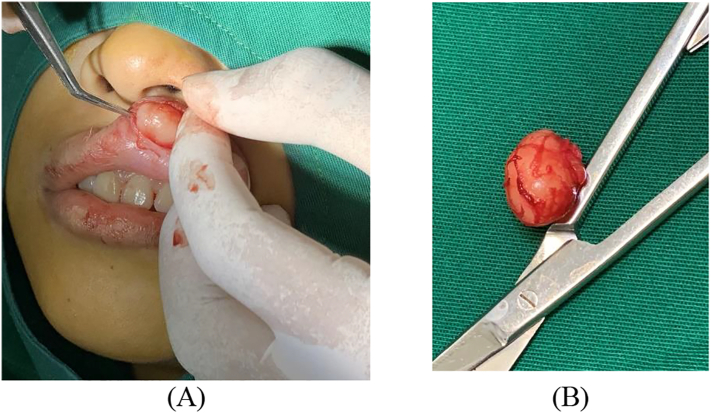
(A) Excision and evacuation of the tumor. (B) Macroscopic appearance of the tumor with the size of 1.5 × 3 cm.

The initial diagnosis of this case was lipoma. The differential diagnosis of mucoceles, hemangiomas, eosinophilic granulomas, epidermoid and dermoid cysts, epithelial hyperplasia, granular cell tumors, and lymphangiomas were made. The patient underwent surgical excision under general anesthesia (Fig. 2) and histopathological anatomy (HPA) examination with hematoxylin-eosin (HE) staining was performed on the tumor tissue.

**Fig. 3:**
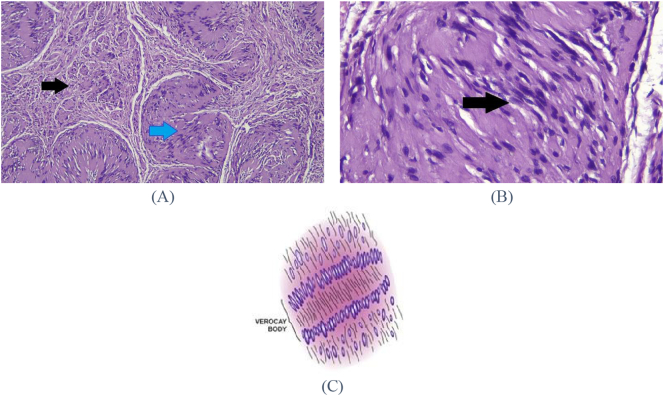
Histological analysis (A) HPA image at 10× magnification, showing monomorphous tumor cells with spindle and wavy oval nuclei appearance, hypocellular areas indicated (Antoni B pattern) by black arrows, and hypercellular areas (Antoni A patterns) indicated by blue arrows. (B) HPA image at 40× magnification showed the arrangement in a palisading pattern of *Verocay* bodies (shown by arrows), a characteristic of Schwannoma. (C) Schematic image of palisading pattern in *Verocay* body.

The HPA image showed two area with different pattern: a hypocellular pattern in *Antoni B* and a hypercellular pattern in *Antoni A* as well as a palisading array of cells forming an image of *Verocay* bodies (Tiger skin appearance) which is a typical histopathological feature of Schwannoma (Fig. 3).

**Fig. 4:**
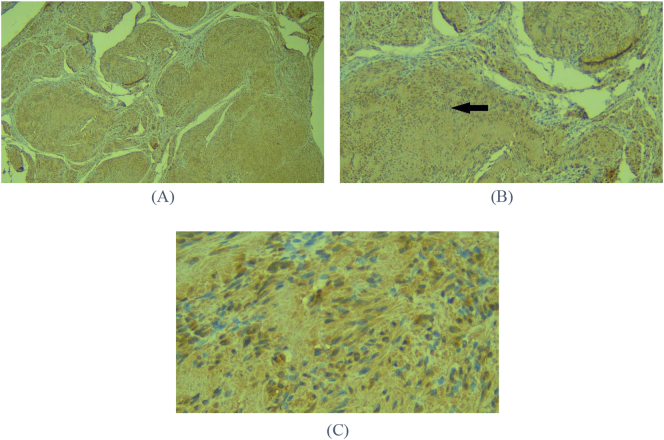
Immunohistochemical analysis of the excised tumor (A) Neoplasm cells (nucleus and cytoplasm) stained positively on all tumor cells with moderate to strong intensity (IHC S100, magnification 40×), (B) neoplasm cells in the Antoni A pattern with positive stain are shown by the arrow. The stained cells indicate S100 protein with strong intensity (IHC S100, magnification 100×), (C) higher magnification showing the neoplasm cells in the Antoni A pattern with positive stain are shown by the arrow. The stained cells indicate S100 protein with strong intensity (IHC S100, magnification 400×).

Immunohistochemical examination (IHC) was performed to confirm the diagnosis. The S100 protein was used in the IHC assay because Schwannoma were highly reactive with S100 protein staining [10]. The IHC examination showed that the entire cytoplasm was stained positively in all tumor cells with a strong intensity (Fig. 4). The brown-stained cytoplasm and the blue-stained nucleus were seen in the IHC results at 400× magnification (Fig. 4). The intensity of staining was stronger in the Antoni A pattern than the Antoni B pattern. These results confirmed the histopathological and clinical diagnosis for Schwannoma.

**Fig. 5:**
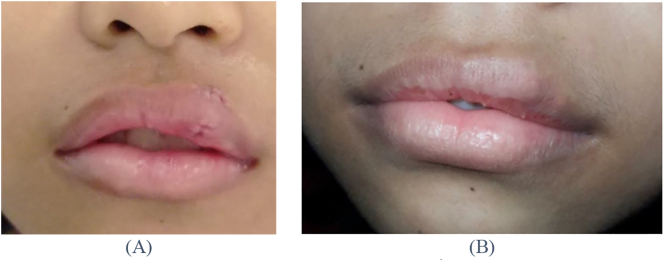
Clinical photograph during the follow-up period. (A) 7th day postoperative follow-up, no dehiscence of the wound, scar was noticed in the operation area, (B) 1-year postoperative follow-up, the wound was completely closed, there was no sign of recurrence, esthetic healing was achieved.

Evaluation at 7th day postoperative showed no dehiscence of the wound, no signs of infection, and minimum degree swelling in the surgical area. All the mucosal and lip sutures were removed. Follow-up of 1 year after surgery showed that the wound was completely healed and there were no signs of recurrence (Fig. 5).

## 3 Discussion

Schwannoma is a benign tumor originating from *Schwann* cells from myelinated nerve fibers. Schwannomas are usually solitary but may also be multiple when associated with neurofibromatosis [11]. Approximately 2 % of Schwannomas are reported to be malignant with distant metastases [[12], [13], [14]]. Schwannomas are rare in the upper lip; because of the anatomy of the lips themselves. Lips are composed of connective tissue, fat, skin, and minor salivary glands; so that the lesions that often occur are lipomas, mucoceles, and fibromas [8].

Several literatures reported different prevalence of Schwannoma of intraoral origin. Studies in Brazil reported that Schwannoma represented only 0.02 %–0.04 % of all intraoral lesion [13,15]. The approximate incidence of intraoral Schwannoma was only 1 % [16]. Based on its location intraorally, 52 % of this lesion occurred in tongue, 20 % in buccal mucosa, 9 % in soft palate, and 19 % in gingiva and lip [4] Other study found that the incidence of Schwannoma was 11.7 % in tongue and pharynx [17].

Until now, there were only 12 cases of Schwannoma of the upper lip and only 4 (four) of them were in adolescent age group (<20 years old) (Table 1). Some literature states that Schwannoma is asymptomatic, but sometimes it can be symptomatic according to the origin of the nerve cells. This tumor is characterized by a slow growing solitary mass with a smooth surface, and the structure of the mass may vary from fluctuating cyst to solid [18]. The clinical presentation from previous reports correspond to the case in this report in which patient has no symptoms, slow growth, solitary mass with a smooth surface, color similar to the surrounding mucosa, and fluctuating solid mass structure [12].Clinically, diascopy test might be useful for distinguishing Schwannoma with hemangioma. Diascopy test involves using glass slide or other clear material to depress the lesion. Dissipating blood intravascularly will give the “blanched” appearance, resulted in positive result [19]. Both lesions exhibited numerous blood vessels and hyalinized vessels that make the lesion clinically similar [20]. Negative result in diascopy test rules out hemangioma in the diagnosis [15].

**Table 1:** Reported cases of upper lip schwannoma.

Author	Year	Number of cases	Gender	Age (years)	Follow up	Recurrent	Antoni features
Barbosa and Hansen	1984	1	Male	36	Unknown	No	Unknown
Asaumi et al. [21]	2000	1	Female	20	Unknown	No	Mixed pattern
Yang and Lin [22]	2003	1	Female	22	2 years	No	Mixed pattern
Yilmaz et al. [23]	2004	1	Female	29	1 year	No	Mixed pattern
Hashiba et al. [24]	2007	1	Female	12	3 years	Yes	Antoni A
Humber et al. [25]	2011	1	Female	82	5 years	No	Mixed pattern
Bayindir et al. [6]	2012	1	Male	15	1 year	No	Antoni A
Krishnan et al. [26]	2014	1	Female	28	8 months	No	Mixed pattern
Hajong et al. [27]	2016	1	Female	14	2 years	No	Mixed pattern
Haigh et al. [28]	2017	1	Female	23	Unknown	Unknown	Mixed pattern
Desai [29]	2019	1	Male	14	Unknown	No	Mixed pattern
Chi et al. [30]	2020	1	Male	41	Unknown	Unknown	Unknown

Schwannoma is classified into seven subtypes based on its histopathological features: classic (*Verocay*), cellular, plexiform, cranial nerve, melanotic, degenerated and granular cell Schwannoma. The pattern observed in HPA examination includes the hypercellular area consisting of nucleus palisade, spindle *Schwan* cell (*Antoni A* pattern) and hypocellular round cell area consisting of small round cells in myxoid stroma (*Antoni B* pattern). Free bands of hyalinized collagen nuclei between rows of nuclei form the *Verocay* body [11,31]. Vascularity is not prominent in this lesion, necrosis and mitotic activity are rare [8].

The mechanism of pathogenesis of Schwannoma is divided into sporadic pathways, NF2 pathways, Schwannomatosis pathways, and Carney's complex pathways [12]. Schwannoma occurs due to a defect in the Neurofibromatosis-2 (NF2) gene that functions to produce a protein merlin that controls the growth of *Schwan* cells [32].

Defects in NF2 cause allele loss, mutation and hypermethylation of the promoter genes (22, 67, 71, 74, 98, 158, 172). Subsequently, this makes NF2 gene lose merlin (an NF2 product), which is structurally identical to ezrin, radixin, and moesin (ERM), a protein involved in linking the cytoskeleton to the membrane and predominantly has tumor suppressor activity [12,33]. Tumors that lose merlin will result in increased expression of integrins, disrupt the spread of extracellular matrix cells and cause increased CD44 expression. Eventually, this will lead to increased cell spreading on the extracellular matrix, increased cell proliferation, angiogenesis, and growth of *Schwan* cells [12].

NF2 defects also interfere with the formation of myelin sheaths in the peripheral nervous system nodes of Ranvier which contain K^+^ and Na^+^. It commonly occurs in peripheral nerves and usually in myelin-coated nerve fibers. Central lesions of the Schwannoma usually arise from sensory nerve roots and intracranial areas of the vestibular branch of the eighth nerve, but it can also arise from the trigeminal nerve in the setting of NF2 [12,34,35]. Our group suspect the Schwannoma in this case was originated from the maxillary nerve, which is a branch of the trigeminal nerve, and develops from the superior labialis nerve [12,14].

Management of Schwannoma is excision of the capsule to avoid recurrence. Schwannoma's prognosis is good, with a low rate of malignant emergence if the tumor is removed completely to its base [8]. Among the twelve Schwannoma of the upper lip (Table 1), only one had recurrency after 3-years follow up [24]. Periodic follow-up is very important for early detection of recurrence. This case was followed for 1 year with satisfactory result and no sign of recurrence. This indicates that the tumor was completely removed to its base during the surgical procedure and esthetic results were achieved.

## 4 Conclusion

Upper lip Schwannoma is a very rare tumor, and it is indistinguishable from other benign soft tissue tumors based on clinical findings. Immunohistochemical analysis is the gold standard for histopathological examination, to confirm rare cases of Schwannoma on the upper lip. Schwannoma should be considered as a differential diagnosis for lumps on the lip with sessile, similar color like surrounding tissue, painless, and movable features.

## Consent

Written informed consent from the patient's guardian was obtained for the purpose of examination and treatment of this case. The patient's guardian also gave consent for publication given the guarantee for anonymity so it would not cause harm to the patient and the family. The copy of the written consent is available for review by request to the corresponding author.

## Ethical approval

The patient had given written consent to the examination and treatment that were performed as well as the publication of this case report article.

## Funding

The examination and treatment in this case were supported by funding of *Badan Penyelenggara Jaminan Sosial Kesehatan* (Indonesian Government National Health Insurance).

## Research registration number

Not applicable.

## Guarantor

Any inquiries regarding this publication are the responsibility of the corresponding author, Cahya Yustisia Hasan.

## CRediT authorship contribution statement

Conceptualization, C.Y.H., Y.B.R., and B.P.S.; data collection and visualization, R.M.; supervision and validation, Y.B.R., B.P.S., and C.Y.H.; original draft preparation, R.M. and M.R.P.; review and editing, R.M., M.R.P., Y.B.R., B.P.S., C.Y.H.; all authors have read and agreed to the published version of the manuscript.

## Declaration of competing interest

Authors declared that there is no conflict of interests associated with this manuscript.
